# Complete Genome Sequence of a Lytic *Siphoviridae* Bacteriophage Infecting Several Serovars of *Salmonella enterica*

**DOI:** 10.1128/genomeA.00943-16

**Published:** 2016-09-29

**Authors:** Rubina Paradiso, Serena Lombardi, Maria Grazia Iodice, Marita Georgia Riccardi, Massimiliano Orsini, Sergio Bolletti Censi, Giorgio Galiero, Giorgia Borriello

**Affiliations:** a Istituto Zooprofilattico Sperimentale del Mezzogiorno, Portici, Italy; b Cosvitec s.c.ar.l., Naples, Italy; c Istituto Zooprofilattico Sperimentale G. Caporale, Teramo, Italy

## Abstract

The bacteriophage 100268_sal2 was isolated from water buffalo feces in southern Italy, exhibiting lytic activity against several subspecies of *Salmonella enterica*. This bacteriophage belongs to the *Siphoviridae* family and has a 125,114-bp double-stranded DNA (ds-DNA) genome containing 188 coding sequences (CDSs).

## GENOME ANNOUNCEMENT

Salmonellosis is distributed across the world, on all continents, affecting many domestic and wild species. Among mammalians, ruminants and swine are particularly affected (1). In cattle (1) and water buffalo (2) in several countries, *Salmonella* is a major cause of calf sickness and death. Salmonellosis is a frequent human intestinal disease and, despite well-established controls, *Salmonella* remains a frequent cause of food poisoning. Moreover, antimicrobial resistance in nontyphoidal *Salmonella* is considered one of the major public health threats related to food-animal production. For this reason, bacteriophages are gaining increasing interest as alternative strategies for bacterial control. In this study, we isolated and characterized the lytic bacteriophage 100268_sal2 from water buffalo feces infecting several serovars of *Salmonella enterica*, including *S. enterica* serovar Enteritidis (host bacterium), and *S. enterica* serovar Typhimurium, monophasic *S*. Typhimurium, *S. enterica* serovar Münster, *S. enterica* serovar Paratyphi B, and *S. enterica* serovar Napoli as additional target serovars. Plating efficiencies (3) were medium (>0.1) on monophasic *S*. Typhimurium and *S*.  Paratyphi B, low (<0.1) on *S*. Typhimurium and *S*. Münster, and scarce (<0.001) on *S*. Napoli.

Phage DNA was purified with the QIAamp DNA minikit, as described by the manufacturer. DNA sequencing was performed using the Ion Torrent PGM platform, yielding a total of 335,919 reads, with an average read length of 239 bp and an average coverage of 2,034×. Quality control and trimming were carried out using in-house-implemented Python scripts, assembly was performed using the SPAdes software (4), and finishing was completed using the DraftDoctor software (version 1.0 CRS4; M. Orsini, unpublished data). Genome annotation was manually curated after a preliminary annotation performed by Prokka (5).

The 100268_sal2 genome consisted of 125,114 bp, with a G+C content of 40.2%. The genome contained a long terminal repeat of 11,238 nucleotides (nt) and 188 predicted coding sequences (CDSs). Of those CDSs, 64 matched identified phage genes, including 27 genes involved in phage physiology, 20 genes involved in phage structure, 14 genes involved in DNA replication and three genes responsible for the lytic activity. The remaining 124 CDSs encoded hypothetical proteins. Moreover, 22 tRNA genes, two of which are likely pseudogens, were identified. No genes associated with lysogeny, toxin production, *Salmonella* virulence, or antibiotic resistance were identified, therefore suggesting a possible use of this phage as a prophylactic agent for the control of *Salmonella*. Further analysis, however, will be required to assign potential functions to the several unidentified and hypothetical genes.

Blast analysis indicated that the bacteriophage 100268_sal2 belongs to the *Siphoviridae* family and showed 90.8% identity (determined with EMBOSS stretcher) to phage Stitch (6) it was therefore classified as a T5-like phage. According to the genome structure of T5 phages (7), the bacteriophage 100268_sal2 exhibits a genome functionally divided into three parts: preearly, early, and late regions. The preearly region includes a large terminally repeated sequence. The early genes are involved in phage metabolism, DNA replication, and lysis, also including a tRNA gene cluster. The late genes encode structural proteins for mature phage particles.

### Accession number(s).

The complete genome of the bacteriophage 100268_sal2 has been deposited in GenBank under the accession number no. KU927497.

## References

[1] Millemann Y, Evans S, Cook A, Sischo B, Chazel M, Buret Y 2010 Salmonellosis, p 947–984. *In* Lefevre PC, Blancou J, Chermette R, Uilenberg G (ed), Infectious and parasitic diseases of livestock, 1st ed. Lavoisier, Paris, France.

[2] Borriello G, Lucibelli MG, Pesciaroli M, Carullo MR, Graziani C, Ammendola S, Battistoni A, Ercolini D, Pasquali P, Galiero G 2012 Diversity of *Salmonella* Typhimurium strains isolated from the intestines of water buffalo calves with gastroenteritis. BMC Vet Res 8:201. doi:10.1186/1746-6148-8-201. 23098237 PMC3514206

[3] Khan Mirzaei M, Nilsson AS 2015 Isolation of phages for phage therapy: a comparison of spot tests and efficiency of plating analyses for determination of host range and efficacy. PLoS One 10:e0118557. doi:10.1371/journal.pone.0118557. 25761060 PMC4356574

[4] Bankevich A, Nurk S, Antipov D, Gurevich AA, Dvorkin M, Kulikov AS, Lesin VM, Nikolenko SI, Pham S, Prjibelski AD, Pyshkin AV, Sirotkin AV, Vyahhi N, Tesler G, Alekseyev MA, Pevzner PA 2012 SPAdes: a new genome assembly algorithm and its applications to single-cell sequencing. J Comput Biol 19:455–477. doi:10.1089/cmb.2012.0021. 22506599 PMC3342519

[5] Seemann T 2014 Prokka: rapid prokaryotic genome annotation. Bioinformatics 30:2068–2069. doi:10.1093/bioinformatics/btu153. 24642063

[6] Grover JM, Luna AJ, Wood TL, Chamakura KR, Kuty Everett GF 2015 Complete genome of *Salmonella enterica* serovar Typhimurium T5-like siphophage Stitch. Genome Announc 3(1):e01435-14. doi:10.1128/genomeA.01435-14. 25657270 PMC4319617

[7] Wang J, Jiang Y, Vincent M, Sun Y, Yu H, Wang J, Bao Q, Kong H, Hu S 2005 Complete genome sequence of bacteriophage T5. Virology 332:45–65. doi:10.1016/j.virol.2004.10.049. 15661140

